# Oxygen

**Published:** 1881-01

**Authors:** 


### OXYGEN.

IS OXYGEN a curative agent? The wonderful power which it posesses of destroying organic matter, and the purifying effect which always results therefrom, has led us, reasoning by analogy, to believe that many diseases which are now regarded as incurable, would succumb to the cleansing power of this element. The air contains only 20.095 per cent. of oxygen, the remaining 79.005 parts being composed almost entirely of nitrogen, which serves to dilute the oxygen. The depressing effect of a smaller amount of oxygen and increased amount of carbonic acid is felt when one has been for a short time in a poorly ventilated room.

In mountainous countries, where the height above the sea level is not too great, the refreshing effect of the air is proverbial. This is simply because the proportion of oxygen is greater and of carbonic acid less.

The purifying effect of oxidation is strikingly shown in running brooks. Here water which is unfit to drink on account of organic impurities, becomes pure by running a mile or two. This change is due to the fact that in the act of flowing each particle of the water is brought into contact with the air, and absorbs the necessary amount of oxygen to combine with the organic matter, thus destroying it.

In many diseases a "change of air" is recommended as a cure, or at least as a source of relief. In its incipient stages consumption may often be cured by vigorous exercise in the open air, and by living wholly out of doors.

The benefit derived from pure air and exercise is, in our opinion, due entirely to the large amount of oxygen which exercise—such as horseback riding—enables and compels the patient to inhale. The organic germs of disease are thus oxidized and destroyed.

We submit, then, to the public for consideration, the question: Will not the breathing of pure oxygen gas prove to be the solution to the problem, "How shall we treat consumption?" There is a well authenticated case in which a child was cured of hydrophobia by inhaling three cubic feet of oxygen. In this case, blood poisoning was the evil, and oxygen seems to have combined with the poisonous principle, thereby destroying it.

A young Frenchman who has recently been experimenting upon himself, finds that he can inhale oxygen without experiencing any ill effects. He took as much as one hundred litres a day for several days. The writer has often inhaled oxygen for experimental purposes, and its use was never followed by any unpleasant effect.

